# The ER Stress-Mediated Mitochondrial Apoptotic Pathway and MAPKs Modulate Tachypacing-Induced Apoptosis in HL-1 Atrial Myocytes

**DOI:** 10.1371/journal.pone.0117567

**Published:** 2015-02-17

**Authors:** Jiaojiao Shi, Qi Jiang, Xiangwei Ding, Wenhua Xu, Dao W. Wang, Minglong Chen

**Affiliations:** Division of Cardiology, The First Affiliated Hospital of Nanjing Medical University, Nanjing, Jiangsu, China; Faculty of Medicine & Health Sciences, UNITED ARAB EMIRATES

## Abstract

**Background and Object:**

Cell apoptosis is a contributing factor in the initiation, progression and relapse of atrial fibrillation (AF), a life-threatening illness accompanied with stroke and heart failure. However, the regulatory cascade of apoptosis is intricate and remains unidentified, especially in the setting of AF. The aim of this study was to explore the roles of endoplasmic reticulum (ER) stress, mitochondrial apoptotic pathway (MAP), mitogen-activated protein kinases (MAPKs), and their cross-talking in tachypacing-induced apoptosis.

**Methods and Results:**

HL-1 cells were cultured in the presence of tachypacing for 24 h to simulate atrial tachycardia remodeling. Results showed that tachypacing reduced cell viability measured by the cell counting kit-8, dissipated mitochondrial membrane potential detected by JC-1 staining and resulted in approximately 50% apoptosis examined by Hoechst staining and annexin V/propidium iodide staining. In addition, the proteins involved in ER stress, MAP and MAPKs were universally up-regulated or activated via phosphorylation, as confirmed by western blotting; and reversely silencing of ER stress, caspase-3 (the ultimate executor of MAP) and MAPKs with specific inhibitors prior to pacing partially alleviated apoptosis. An inhibitor of ER stress was applied to further investigate the responses of mitochondria and MAPKs to ER stress, and results indicated that suppression of ER stress comprehensively but incompletely attenuated the activation of MAP and MAPKs aroused by tachypacing, with the exception of ERK1/2, one branch of MAPKs.

**Conclusions:**

Our study suggested tachypacing-induced apoptosis is regulated by ER stress-mediated MAP and MAPKs. Thus, the above three components are all promising anti-apoptotic targets in AF patients and ER stress appears to play a dominant role due to its comprehensive effects.

## Introduction

The underlying mechanisms responsible for atrial fibrillation (AF) are multifactorial. Cardiomyocyte apoptosis, the primary stage of fibrosis, has been approved to be substantially relevant to the occurrence, development and prognosis of AF [1–5]. Mitochondria play a central role in integrating apoptotic pathways and implement function via disruption of the mitochondrial membrane potential (△ψm) and release of cytochrome c, both of which are controlled by the evolutionarily conserved B-cell lymphoma-2 (BCL-2) family proteins [6]. Endoplasmic reticulum(ER) stress, triggered as an adaptive process, has been reported to have a great impact on apoptosis. The protective function of ER stress relies on accelerated unfolded protein degeneration, enhanced protein folding capacity and inhibited translation of mRNAs which constitute the so-called unfolded protein response (UPR), which activated by Grp78 release and subsequent transmembrane proteins (PERK, IRE1, and ATF6) aggregation. If severe ER stress cannot be mitigated, cell apoptosis will be promoted as the last resort to remove dysfunctional cells. Multiple mechanisms involving calcium, caspases, transcription factors, and Bcl-2 family proteins have been proposed for linking the severe ER stress to apoptosis [7]. Mitogen activated protein kinases (MAPKs) composed of extracellular signal-regulated kinase p44/42 MAPK (ERK1/2), p38 MAPK (p38), and c-Jun N-terminal kinase (JNK) have also been regarded as important components of apoptotic regulation and respondents to ER stress [8–10]. However, few systematic research regarding interactions among the above-mentioned pathways acting on apoptosis has been reported, especially in AF model.

Identifying the mechanisms of apoptosis in AF is appealing. In this study, we established cell model for atrial tachycardia remodeling and testified the effect of 8 Hz pacing load on HL-1 cells for 24 h on the following activities: (1) apoptosis in HL-1 cells, (2) activation of ER stress, mitochondrial apoptotic pathway (MAP), MAPKs pathway and their biological functions in apoptosis, and (3) cross-talking between the above three pathways.

## Materials and Methods

### Reagents

Antibodies and other reagents are described in the Supporting Information (S1 File).

### HL-1 cell culture and tachypacing

HL-1 cells were kindly provided by Dr. William Claycomb (Louisiana State University Health Science Center, New Orleans, LA, USA) [11,12] and were cultured in supplemented Claycomb Medium (Sigma–Aldrich, St. Louis, Mo). The spontaneous beating rate of confluent HL-1 cells (72–96 hours after passage) observed at room temperature in our study was 0.6Hz on average. HL-1 cells were starved in serum-free medium for 24 h from the 2^nd^ day after passage and then subjected to field stimulation. Cultures without stimulation were also performed in parallel. Pacing was administered using a C-Pace100TM-culture pacer and CDish100TM culture dishes (IonOptix Corporation, Netherlands) with a 5-ms pulse width and 8-Hz frequency, selected according to previous studies and our preliminary work [13,14]. The capture efficiency was > 90% according to microscopic examination and shortening of action potential duration (APD) was as previously described (S1 Fig.) [15]. HL-1 cells were treated with Ac-DEVD-CHO (20 μM), SB203580 (10 μM), SP600125 (20 μM), PD98059 (10 μM) or 4-PBA (20 μM), 2 h prior to pacing, to inhibit Caspase-3, P38, JNK, ERK1/2 and ER stress respectively.

### Cell viability

Cell viability was detected by the cell counting kit-8 (CCK-8) (Dojindo, Kumamoto, Japan). Cells were seeded into 6-well plates at 5 × 10^4^ cells per well, which differed from previous studies in which cells were seeded into 96- well plates. This distinction was due to the pacing device, which is designed only for 6-well plates. After 24 h starvation, cells were exposed to tachypacing for 1, 6, 12, 24 h. A total of 150 μl CCK-8 was added to each well and incubated at 37°C for 2 h and then the absorbance at 450 nm was measured with a microplate reader (BioTek, USA).

### Hoechst 33342 staining

Typical morphological features of apoptotic cells were evaluated by Hoechst 33342 staining (Beyotime Institute of Biotechnology, China). After 24 h tachypacing, the cells were washed twice with cold PBS and fixed in freshly prepared 4% paraformaldehyde for 30 min. Cells were then washed with cold PBS again before incubated with 5 μg/ml Hoechst 33342 for 15 min at 37°C in the dark. Finally, cells were washed with PBS and apoptotic cells were identified with fluorescence microscope (Nikon, Tokyo, Japan). Normal cells showed homogeneous blue chromatin with organized structure. By contrast, apoptotic cells presented bright blue chromatin that is highly condensed or fragmented.

### Flow cytometry

Cell apoptosis was also assessed by flow cytometry. After tachypacing, approximately 1× 10^6^cells were harvested, washed twice with pre-chilled PBS, resuspended in 300ml binding buffer and then incubated with annexin V / propidium iodide (PI) (BD PharMingen, San Diego, CA, USA) for 20 min in the dark. The samples were analyzed using a FACScan flow cytometer (BD Biosciences, USA) within 1 h.

### JC-1 staining

△ψm was examined via JC-1 staining (Beyotime Institute of Biotechnology, China). After tachypacing, HL-1 cells were incubated with JC-1 working solution at 37°C in the dark for 20 min and observed by fluorescence microscopy. In healthy cells, where mitochondrial potentials remain depolarized, JC-1 forms complexes of J-aggregates showing punctate red fluorescence at 590nm emission wavelength; however in apoptotic cells, JC-1 remains in the monomeric form showing diffused green fluorescence at 530nm emission wavelength.

### Mitochondrial and cytosolic separation

After 24-h of pacing, HL-1 cells were washed twice with cold PBS and treated with lysis buffer containing protease inhibitors. Then, we collected the buffer and centrifuged for 10 min at 750 × g at 4°C, and the sediment containing the nuclei and unbroken cells was discarded. The supernatant was then centrifuged at 15,000 × g for 15 min. The resulting supernatant was removed and used as the cytosolic fraction. The sediment containing the mitochondria was further incubated with PBS containing 0.5% Triton X-100 for 10 min at 4°C. After centrifugation at 16,000 × g for 10 min, the supernatant was collected as the mitochondrial fraction.

### Western blotting

The protocol of western blotting analysis was as previously described [14].

### Statistical analysis

All results are presented as mean ± standard deviation (SD). Statistical analysis was performed using one-way analysis of variance (ANOVA) with the Student-Newman-Keuls post hoc test for multiple group comparisons. P < 0.05 was considered statistically significant.

## Results

### Tachypacing reduced cell viability in HL-1 cells in a time-dependent manner

**Fig 1:**
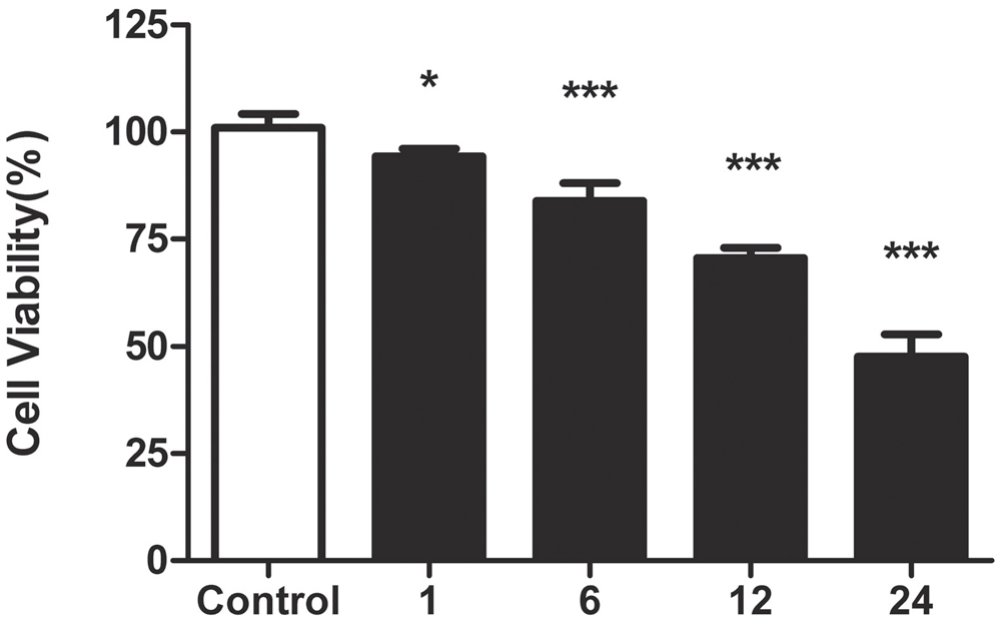
Effects of tachypacing on cell viability. HL-1 cells were subjected to tachypacing for 1, 6, 12 and 24 h. Cell viability was measured with CCK-8 assay and the results are presented as the means ± SD of 3 independent experiments. *P < 0.05; * * *P < 0.001 versus the control group.

Pacing parameters which are pre-defined based on our previous work served to imitate atrial tachycardia remodeling. HL-1 cells were exposed to increasing pacing times of 1, 6, 12 and 24 h, which correlated with decreasing cell viability as indicated in Fig. 1. 24-h pacing load giving rise to 53% reduction of cell viability on average was chosen for the following experiment.

### Tachypacing induced apoptosis in HL-1 cells

**Fig 2:**
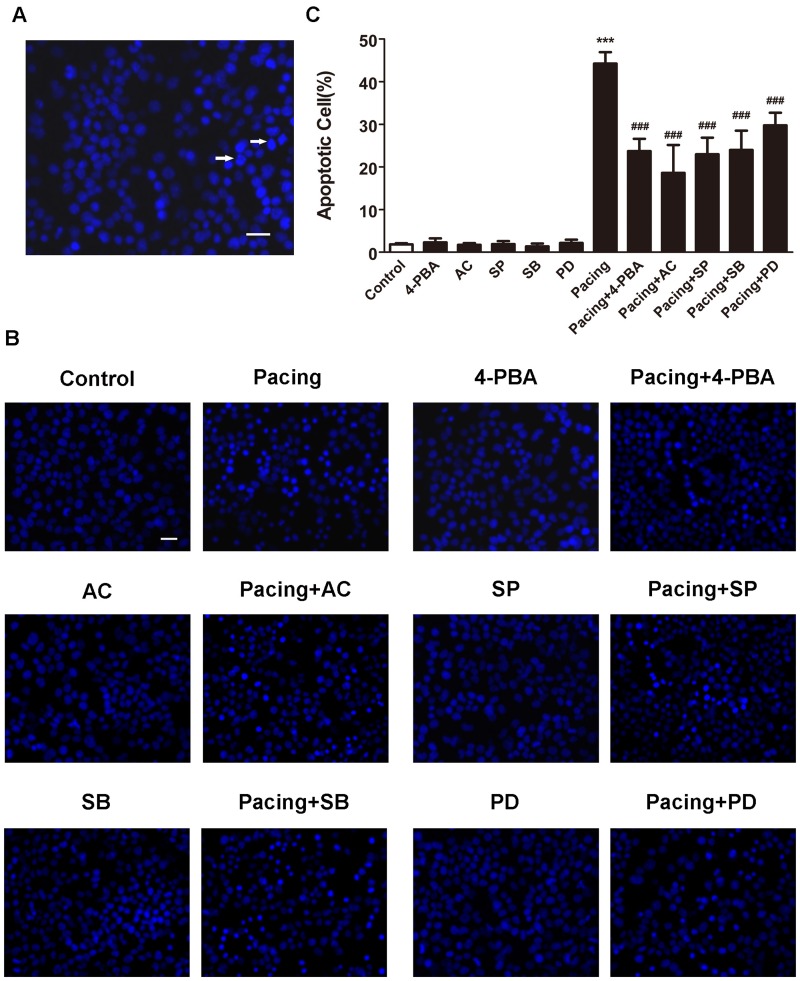
Apoptosis detected by Hoechst 33342 staining. Hoechst-positive cells were observed under a fluorescence microscope. Scale bar = 100 μm. (A) Normal cells displayed intact nuclei and adqulis chromatin. Typical morphological features of apoptosis induced by 24-h tachypacing, such as shrunken cells with condensed or fragmented nuclei, are indicated by arrows. (B) Apoptosis was induced by pacing with or without pretreatment with specific inhibitors 2 h prior to 24-h pacing. (C) The percentage of apoptotic cells is expressed as the means ± SD of 3 independent experiments. * * *P < 0.001 versus the control group; ^# # #^ P < 0.001 versus the pacing group.

**Fig 3:**
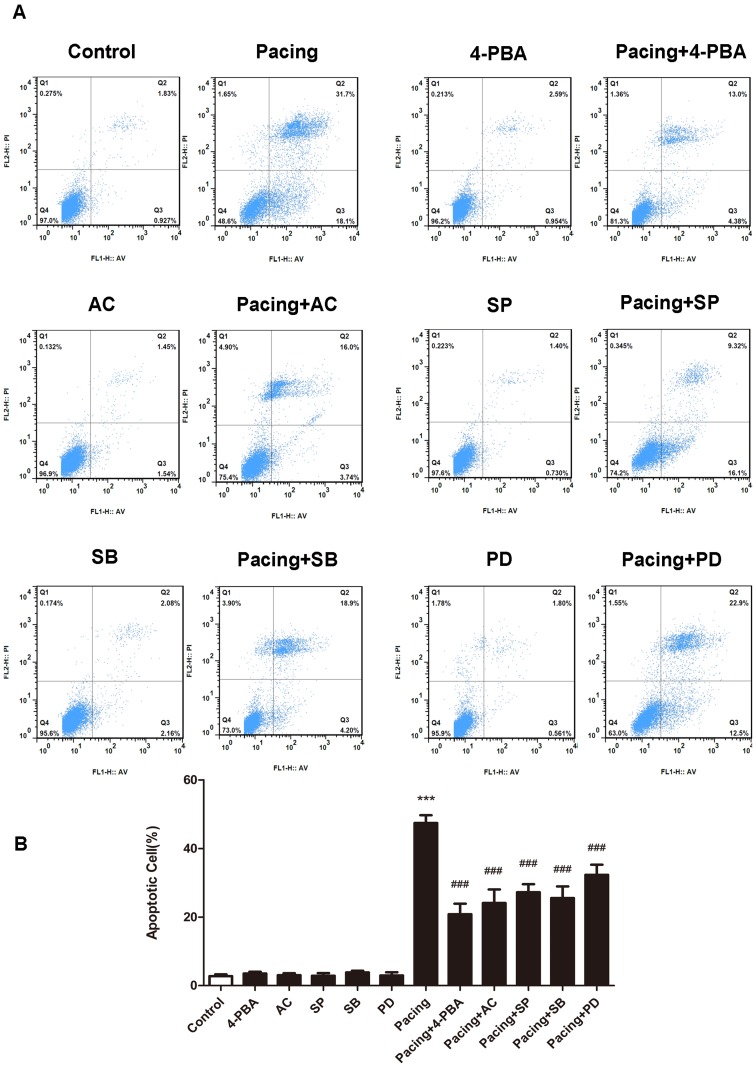
Apoptosis examined by flow cytometry. HL-1 cells were pretreated as indicated. Then, (A) cells were treated with annexin-V/PI double staining and observed by flow cytometry. The data correspond to the percentage of early (AN﹢P﹣) and late (AN﹢PI﹢) apoptotic cell populations. (B) The percentage of apoptotic HL-1 cells is presented as the mean ± SD of 3 independent experiments. * * *P < 0.001 versus the control group; ^# # #^ P < 0.001 versus the pacing group.

After 24 h exposure to tachypacing, HL-1 cells exhibited typical morphological features of apoptosis as condensed or fragmented nuclei (Fig. 2A). The percentage of Hoechst-positive cells in the pacing group was 44.23±2.69% which was significantly higher than 1.84±0.25% in the control group (Fig. 2B and 2C). Similar results were obtained by annexin V/PI staining (Fig. 3A). The apoptotic ratio was 47.43±2.26% in the pacing group *versus* 2.77±0.51% in the control group (Fig. 3B). Accordingly, tachypacing induced apoptosis in HL-1 cells.

### ER stress was involved in tachypacing-induced apoptosis and inhibition of ER stress partially relieved apoptosis

**Fig 4:**
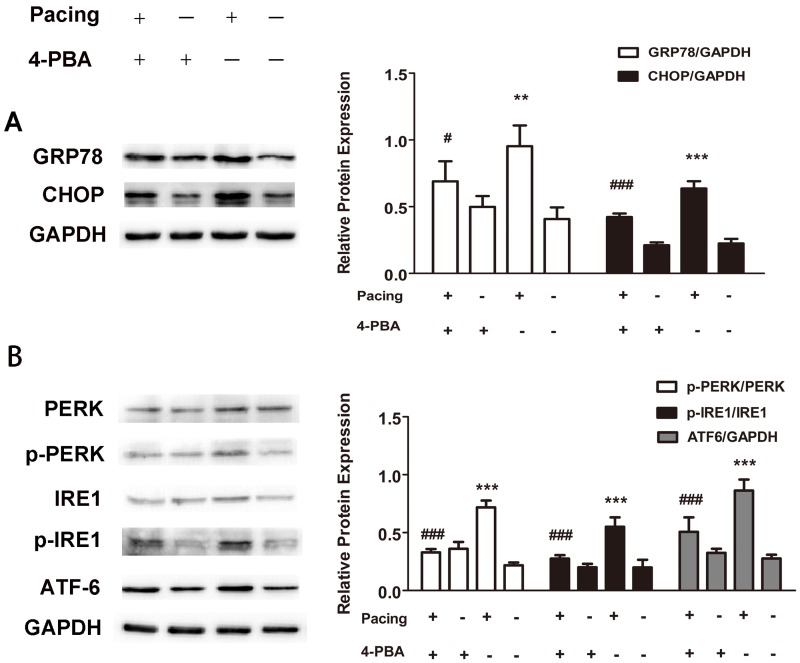
Activation of ER stress followed by pacing-induced apoptosis. Tachypacing was exerted on HL-1 cells for 24 h in the presence or absence of 4-PBA, an inhibitor of ER stress. Protein levels were measured by western blot analysis. GAPDH was used as an internal control. (A) UPR induction presented as elevated GRP78 and CHOP protein level. (B) Western blotting bands of p-PERK, p-IRE1 and ATF6. p-PERK and p-IRE1were normalized to PERK and IRE1 respectively. The quantitative analysis of relative protein level is expressed as the means ± SD of 3 independent experiments. * *P < 0.01 and * * *P < 0.001 versus the control group; ^#^ P < 0.05 and ^# # #^ P < 0.001 versus the pacing group.

To determine whether ER stress takes effects on apoptosis following tachypacing, the protein expression of several ER stress markers was examined by western blotting. The release of ER chaperon GRP78 and enhanced expression of CHOP are generally supposed to be associated with the occurrence and exacerbation of ER stress. In the present study, the protein levels of both GRP78 and CHOP were markedly elevated after 24 h tachypacing compared to the control group, whereas this increase was partially diminished by pretreating with 20μM 4-PBA, an ER stress inhibitor (Fig. 4A). We further assessed three transducers (PERK, IRE1 and ATF6) of ER stress to confirm the pathway involved in pacing-induced ER stress. The results illustrated in Fig. 4B demonstrated that all three of the transducers were activated after tachypacing, as indicated by higher levels of phospho-PERK, phospho-IRE1 and ATF6 protein in comparison to the control group. Similarly, such activation was attenuated by 4-PBA. In addition, the percentage of Hoechst-positive cells was 44.23±2.69% in the pacing group but reduced to 23.70±2.91% in the pacing+4-PBA group (Fig. 2C), which was consistent with the results from annexin V/PI staining showing that the apoptotic ratio decreased to 20.83±3.08% from 47.43±2.26% after 4-PBA pretreatment (Fig. 3B).

Taken together, these results suggest that all three of the ER stress pathways enroll in tachypacing-induced apoptosis and inhibition of ER stress could alleviate apoptosis to a certain degree.

### MAP and caspases activation played roles in tachypacing-induced apoptosis with the control of ER stress

**Fig 5:**
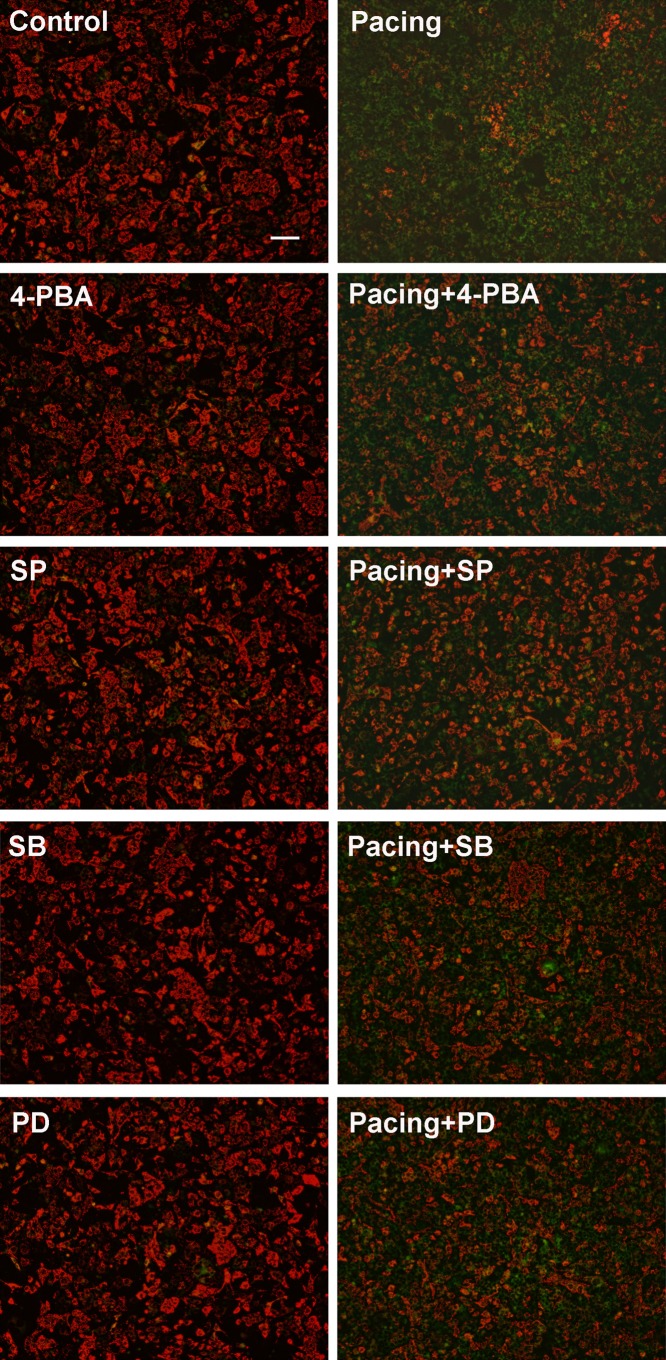
Mitochondrial membrane potential assessed by JC-1 dye. Cells were treated as labeled, stained with JC-1 dye and then observed under a fluorescence microscope. Scale bar = 400μm. Red fluorescence represents JC-1 aggregates formed in normal cells with high △ψm, whereas green fluorescence represents JC-1 monomers in cells with low △ψm.

**Fig 6:**
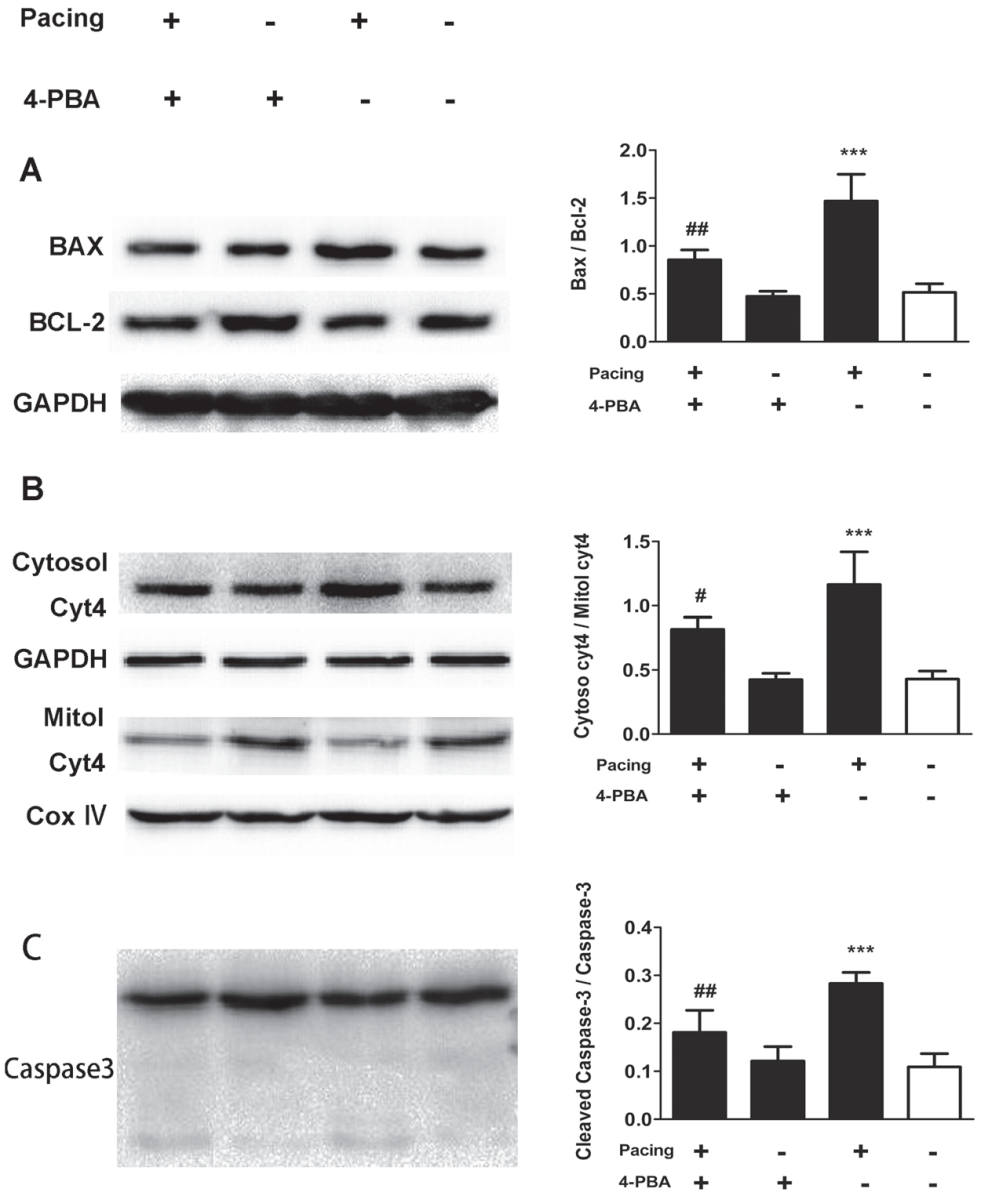
The role of ER stress in the activation of MAP and caspases in pacing-induced apoptosis. HL-1 cells were treated as noted. The protein levels of Bax, Bcl-2(A), cytochrome c (B) and caspase-3 (C) were evaluated by western blot analysis. GAPDH was used as an internal control. Cytochrome c in mitochondria was normalized to COX4. The quantitative analysis of relative protein level is expressed as the means ± SD of 3 independent experiments. * * *P < 0.001 versus the control group; ^#^ P < 0.05 and # # P < 0.01 versus the pacing group.

To elucidate the role of the MAP in tachypacing-induced apoptosis, we made use of JC-1 staining to evaluate the loss of △ψm by fluorescence microscope and western blotting to assess related protein expression. The feeble △ψm is the primary change of cell apoptosis. The results of the JC-1 assay showed that comparing to the control group, △ψm in the pacing group was notably compromised, presenting as a large area of green fluorescence (Fig. 5). With regard to protein expression (Fig. 6), the protein level of pro-apoptotic Bax was elevated accompanying with declining level of anti-apoptotic Bcl-2 in the pacing group compared to the control group. The ratio of cytochrome c in cytosol to that in mitochondria was significantly higher in the pacing group because of the release of cytochrome c from mitochondria to cytosol. The protein level of cleaved caspase-3, a terminal executor of apoptosis, was also elevated in the pacing group, showing roughly 2-fold increase in comparison to the control group. Pretreating with 20 μM ACDEVD-CHO, a caspase-3 inhibitor, protected HL-1 cells against apoptosis. The results in Fig. 2C showed the percentage of Hoechst-positive cell in pacing group was 44.23±2.69% while in the pacing+ACDEVD-CHO group, the ratio reduced to 18.59±6.58%. Identical results were revealed by annexin V/PI staining.

4-PBA was utilized to assess the influence of ER stress on MAP. As expounded in Fig. 6, compared to the pacing group, over-expression of the above-mentioned proteins tended to be moderated in the pacing+4-PBA group although it was still higher than that in the control group.

Thus, the ER stress-regulated MAP plays an important role in tachypacing-induced apoptosis and inhibition of ER stress broadly eases the high expression of proteins relevant to the MAP induced by tachypacing.

### MAPKs pathway engaged in pacing-induced apoptosis adjusted by ER stress

**Fig 7:**
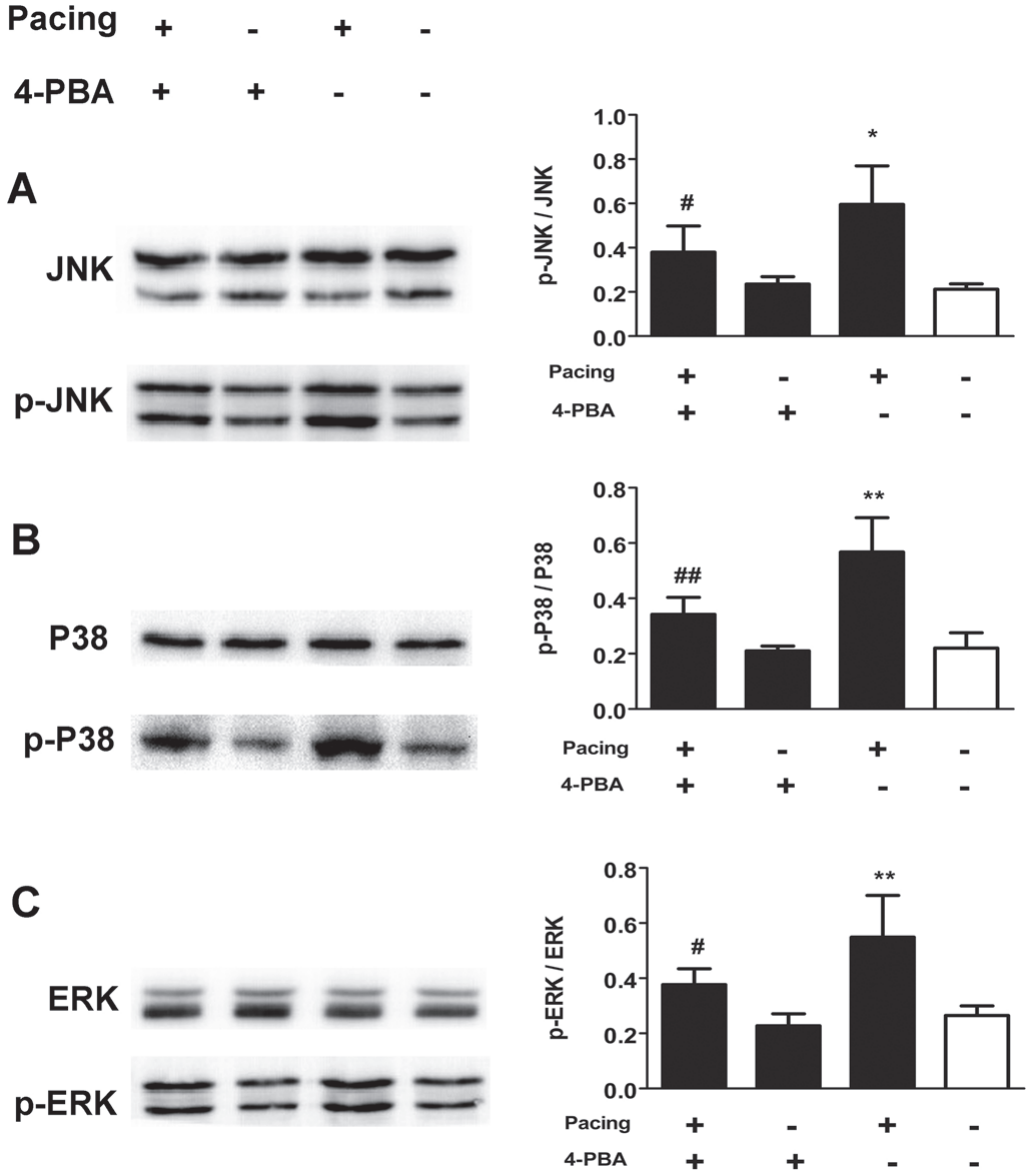
The impact of ER stress on the phosphorylation of MAPKs in pacing-induced apoptosis. HL-1 cells were treated as labeled. The protein levels of phosphorylated JNK (A), P38 (B) and ERK (C) were normalized to total JNK, P38 and ERK. The quantitative analysis of relative protein level is expressed as the means ± SD of 3 independent experiments. *P < 0.05 and * * P < 0.01 versus the control group; ^#^ P < 0.05 and ^# #^ P < 0.01 versus the pacing group.

To validate the involvement of MAPKs in tachypacing-induced apoptosis, we first examined the phosphorylation level of MAPKs via western blotting. The results in Fig. 7 showed that 24 h tachypacing brought about distinct increases in phosphorylation level of JNK, p38 and ERK1/2, in comparison with the control group. To make a deeper insight into the function of MAPKs in tachypacing-induced apoptosis, we applied 10 μM SP600125, 10 μM SB203580, and 20 μM PD98059, inhibitors of JNK, p38 and ERK1/2 respectively, prior to pacing. As illustrated in Fig. 2, Fig. 3 and Fig. 5, MAPKs inhibitors not only reversed the deteriorated △ψm to varying degree but also led to reductions of apoptosis ranging from 15% to 20% calculated by Hoechst 33342 staining and annexin V/PI staining.

We made use of 4-PBA to evaluate the impact of ER stress on MAPKs. The results in Fig. 6 presented that pretreatment with 4-PBA lessened the phosphorylation of JNK and p38. Nevertheless, the phosphorylation level of ERK1/2 in the pacing group was not statistically distinct from that in the pacing+4-PBA group.

Our results show that ER stress-supervised MAPKs pathway participates in tachypacing-induced apoptosis. Moreover, inhibition of ER stress in advance prevents the excessive phosphorylation of JNK and p38 MAPK but ERK1/2 aroused by tachypacing.

## Discussion

The current study was designed to make a further extension of the regulatory mechanisms of apoptosis on the basis of cell model for atrial tachycardia remodeling. The main implications of this study are as follows: 1. tachypacing can induce apoptosis in HL-1 cells; 2. ER stress, MAP and MAPKs collaboratively modulate apoptosis induced by tachypacing and 3. MAP and MAPKs achieve their functions under ER stress mediation.

Substantial evidence confirms that HL-1 atrial myocytes subjected to tachypacing possess important phenotypic characteristics of atrial tachycardia remodeling similar to AF, such as shortening of APD and down-regulation of L-type Ca^2+^ currents [13,15–17]. In our study, we detected cell apoptosis after 8-Hz tachypacing for 24 h, which was consistent with the pathological process of AF patients.

To date, a large body of evidences has proven that ER stress contributes to apoptotic regulation in the setting of neurodegeneration, heart failure and diabetes; yet the systematic research revealing the role of ER stress in apoptosis within AF model is few. Our study showed that after 24 h pacing, the protein levels of GRP78, CHOP, p-PERK, p-IRE1 and ATF6 were simultaneously elevated to varying degrees, whereas suppression of ER stress can incompletely modify these elevations. However, the explicit biological function of ER stress on apoptosis might differ owing to the different background. Some studies considered that down-regulation of apoptosis was induced by restraining ER stress in Alzheimer disease, heart failure and diabetes [18–20]. By contrast, another study showed that ER stress facilitated the survival of LPS-induced HL-1 cells by promoting autophagy [21]; Interestingly, there is also a study showing that knock-down of CHOP, a crucial element of ER stress, reduced cell survival [22]. Our findings indicated that dissipation of △ψm was partly ameliorated and relaxation of apoptosis could be generated by ER stress inhibitor, which appears to comply with the former findings. Actually, this contradiction could make sense. ER stress essentially has both pro-survival and pro-apoptotic functions, the selection of which depends on the severity and duration of the ER stress. For example, transient activation of IRE1 can cleave and splice the X-box binding protein (XBP1), promoting ER homeostasis by up-regulating of ER-associated degradation (ERAD) components as well as molecular chaperones and enzymes that fold misfolded proteins. Once the ER stress prolongs, IRE1 makes downstream JNK to be phosphorylated, leading to apoptosis.

**Fig 8:**
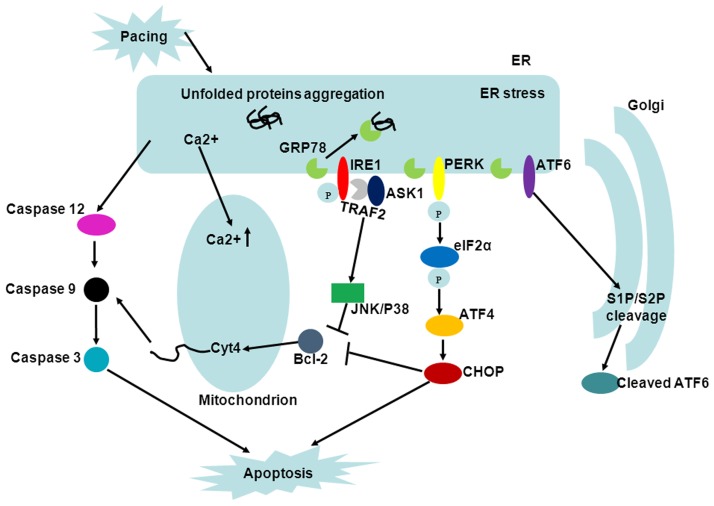
UPR and cross-talking among ER stress, MAP and MAPKs in apoptosis. Three transducers (PERK, IRE1 and ATF6) of ER stress are depicted. JNK and P38 are phosphorylated mainly through the IRE1-TRAF2-ASK1-MKK4/7 pathway and the pathway of ERK1/2 remains challenging. MAP can be intensified because of the elevated Ca ^2+^ concentration in mitochondria, over-expression of Bcl-2 and activated caspase-12, which are induced by ER stress.

Mitochondria are not only the major energy producer of the cells, but also the governor of apoptosis in the final stage. The apoptotic regulation of mitochondrial is realized mainly through BCL-2 family proteins. In our study, the protein levels of pro-apoptotic Bax raised while anti-apoptotic Bcl-2 declined after 24 h pacing. Disproportionality of Bax/Bcl-2 enables permeabilization of the outer mitochondrial membrane, thereby releasing cytochrome c to cytosol and ultimately activating caspases-3, both of which were reflected in our study as an elevated ratio of cytochrome c in cytosol/ mitochondria and expression level of cleaved caspase-3. Moreover, inhibition of caspase-3 notably attenuated the apoptosis induced by tachypacing. Increasing evidences points out a close communication between the MAP and ER stress and here we discovered that application of ER stress inhibitor broadly allayed activation of MAP. Hence, we proposed potential cross-talking as follows (Fig. 8): 1. The excessive ER stress-caused Ca ^2+^ overload in mitochondria is an advantage of mitochondrial membrane permeabilization and subsequent release of cytochrome c [23]; 2. The PERK-eIF2α-ATF4-CHOP pathway of ER stress could directly down-regulate anti-apoptotic Bcl-2 protein [24]; 3. caspase-12, the hallmark of ER stress can successively activate caspase-9 and caspase-3 independently of cytochrome c [25].

MAPKs, a group of evolutionarily conserved Ser/Thr protein kinases, play a dominant role in gathering, amplification and transmission of signals. The diverse biological functions of MAPKs are dependent on a variety of factors such as the subcellular localization, the magnitude and duration of MAPKs activation and interaction with other pathways. In general, JNK and P38 MAPK mainly cope with stress and cellular damage, whereas ERK1/2 regulates cellular differentiation and proliferation [26]. In our study, we testified phosphorylation level of MAPKs and the results implied that JNK, P38 and ERK1/2 are comprehensively activated following tachypacing. To further identify the biological function of MAPKs in the context of rapid pacing, specific inhibitors of MAPKs were applied ahead of tachypacing. We found that inhibition of JNK and P38 could amend both the dissipation of △ψm and apoptosis and these results are accordant with previous studies demonstrating that JNK and P38 are pro-apoptotic [27,28]. Nevertheless, it is confounding that inhibition of ERK1/2, which is generally thought to be pro-survival primarily via BCL-2 family proteins, produced similar results with the two formers, although there are indeed some studies validating that sustained activation of ERK1/2 different from transient activation is pro-apoptotic [29,30]. The concrete mechanisms accounting for totally opposite prognosis may be intricate and partly due to the different stimuli and duration of ERK1/2 activation. It is documented that MAPKs participate in ER stress. In ER stress, JNK and P38 are phosphorylated principally via the IRE1-TRAF2-ASK1-MKK4/7 pathway (Fig. 8) and the phosphorylation of ERK1/2 is also proposed to be IRE1-related, but the precise transduction cascade has not been specified [31]. To address this issue, we utilized 4-PBA to identify the response of MAPKs to ER stress. The results revealed that suppression of ER stress could decrease the elevated phosphorylation level of JNK and p38 caused by pacing but not that of ERK1/2. This apparent discrepancy, taking into account various ERK1/2-related pathways responsible for proliferation or differentiation, may be because inhibition of ER stress alone is not sufficient to detectably assuage phosphorylation of ERK1/2. In addition, JNK and P38 have also been reported to actively influence ER stress [32,33]. Evidently, a large amount of work has yet to be done to integrate regulatory mechanisms between ER stress and MAPKs.

In summary, the results of our study indicate that ER stress, MAP and MAPKs are all promising anti-apoptotic targets in AF patients and ER stress appears to play a dominant role due to its’ comprehensive effects.

## Limitation

HL-1 atrial myocytes stimulated for 24 h is a validated cellular model for atrial tachycardia remodeling. However, the cell culture conditions can not completely replicate the in vivo environment. Further animal experiment and human study are necessary to validate our results.

## Supporting Information

S1 Fig Effect of pacing on action potential duration (APD) in HL-1 myocytes. APD was recorded at room temperature. (A) Representative action potential recording at indicated stimulation frequency is shown in HL-1 cells. The control group was cultured with no tachypacing (0 Hz). (B) Action potential duration was compared at 90% of repolarization (APD90) among conditions. The results are presented as the means ± SD of 3 independent experiments. * *P < 0.01 and * * *P < 0.001 versus the control group.
(TIF)

S1 File (DOCX)
