# Supplementary figures and images for: The ER Stress-Mediated Mitochondrial Apoptotic Pathway and MAPKs Modulate Tachypacing-Induced Apoptosis in HL-1 Atrial Myocytes
Source: PLoS One. 2015 Feb 17;10(2):e0117567. doi: 10.1371/journal.pone.0117567 (PMC4331367; doi:10.1371/journal.pone.0117567)

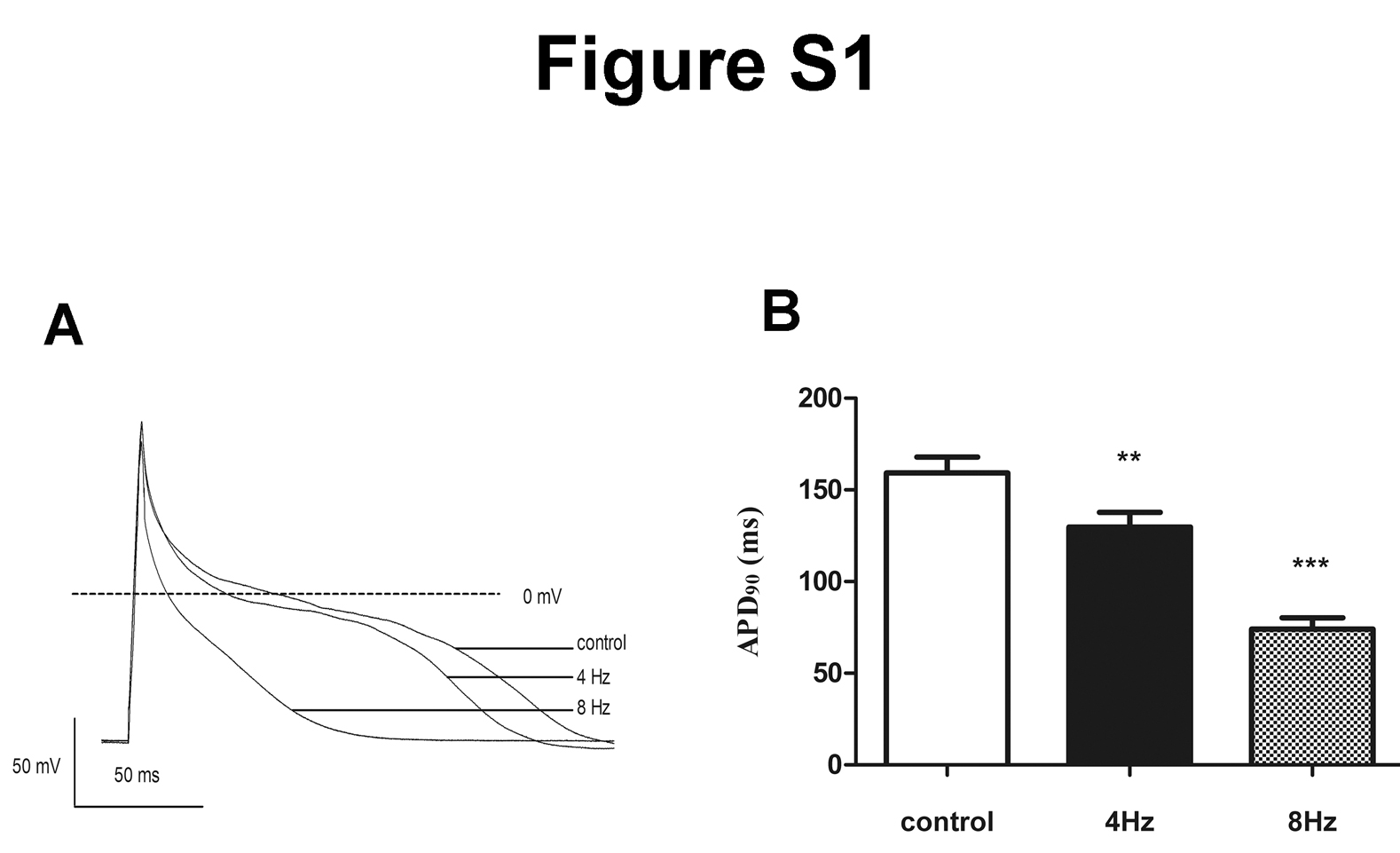

Supplement: S1 Fig — APD was recorded at room temperature. (A) Representative action potential recording at indicated stimulation frequency is shown in HL-1 cells. The control group was cultured with no tachypacing (0 Hz). (B) Action potential duration was compared at 90% of repolarization (APD90) among conditions. The results are presented as the means ± SD of 3 independent experiments. * *P < 0.01 and * * *P < 0.001 versus the control group. (TIF) [file pone.0117567.s001.tif]
