# Supplementary material for: The ER Stress-Mediated Mitochondrial Apoptotic Pathway and MAPKs Modulate Tachypacing-Induced Apoptosis in HL-1 Atrial Myocytes
Source: PLoS One. 2015 Feb 17;10(2):e0117567. doi: 10.1371/journal.pone.0117567 (PMC4331367; doi:10.1371/journal.pone.0117567)
Supplement: S1 File — (DOCX) [file pone.0117567.s002.docx]

**Supporting Information**

**Reagents**

Antibodies targeting the 78-kDa glucose-regulated protein (GRP78), CCAAT/enhancer-binding protein-homologous protein/growth arrest and DNA damage-inducible gene 153 (CHOP), PKR-like ER kinase (PERK), Bax, Bcl-2, caspase-3, cytochrome c, cytochrome c oxidase subunit IV (COX4), SAPK/JNK, phospho-SAPK/JNK(Thr183/Tyr185), p38 MAPK, phospho-p38 MAPK (Thr180/Tyr182), p44/42 MAPK (Erk1/2), phospho-p44/42 MAPK (Erk1/2) (Thr202/Tyr204) and GAPDH were purchased from Cell Signaling Technology (Danvers, MA, USA). Antibodies targeting inositol-requiring enzyme 1 (IRE1), phospho-IRE1 and activating transcription factor 6 (ATF6) were purchased from Abcam Ltd (USA). Phospho-PERK was purchased from Santa Cruz Biotechnology, SB203580 (p38 MAPK inhibitor), Ac-DEVD-CHO (Caspase-3 inhibitor), SP600125 (JNK inhibitor), PD98059 (ERK1/2 inhibitor) and 4-phenylbutyric acid (4-PBA) (ER stress inhibitor) as well as all other chemical reagents, unless otherwise stated, were purchased from Sigma (St. Louis, MO, U.S.A.).

**Electrophysiological Recordings**

Stimulated myocytes were paced using a C-Pace100TM-culture pacer (IonOptix Corporation, Netherlands) with 5-ms duration and 8-Hz square-wave pulses for 24 h. Capture efficiency > 90% was confirmed by microscopic examination and by shortening of the action potential duration (Figure S1). Action potentials were measured in current-clamp conditions at room temperature as previously described [[1](#_ENREF_1),[2](#_ENREF_2)], and they were elicited by 2.5-ms square current pulses (intensity 0.75 nA) at a rate of 8 Hz and sampled at 10 kHz. Data were acquired using a patch–clamp amplifier (Axopatch 200B, Molecular Devices Inc., Silicon Valley, CA) connected to a Digidata 1440A interface (Molecular Devices Inc.)

**References**

1. Brundel BJ, Kampinga HH, Henning RH (2004) Calpain inhibition prevents pacing-induced cellular remodeling in a HL-1 myocyte model for atrial fibrillation. Cardiovasc Res 62: 521-528.

2. Yang Z, Shen W, Rottman JN, Wikswo JP, Murray KT (2005) Rapid stimulation causes electrical remodeling in cultured atrial myocytes. J Mol Cell Cardiol 38: 299-308.

**
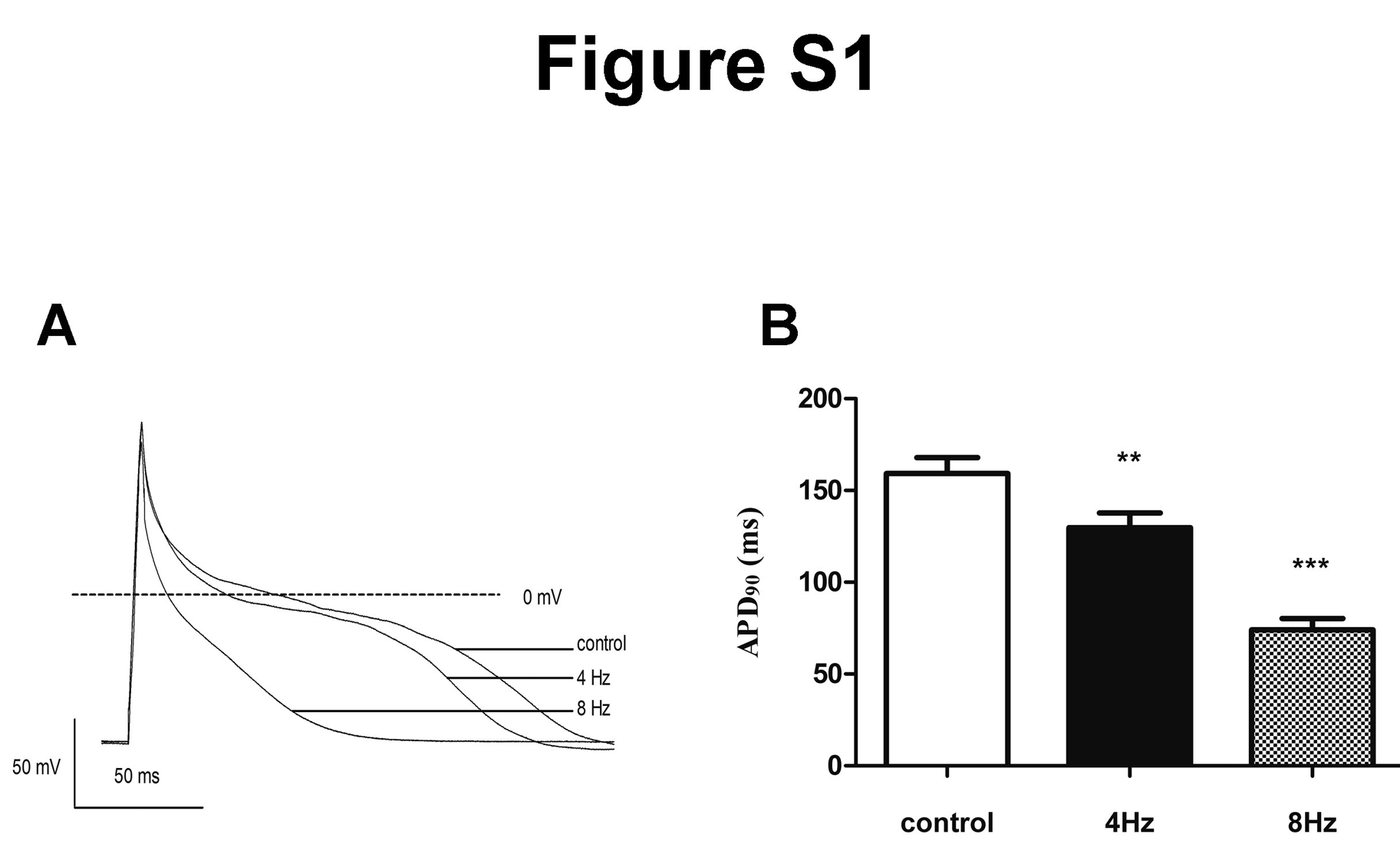
**

**Figure S1. Effect of pacing on action potential duration (APD) in HL-1 myocytes:** APD was recorded at room temperature. (A) Representative action potential recording at indicated stimulation frequency is shown in HL-1 cells. The control group was cultured with no tachypacing (0 Hz). (B) Action potential duration was compared at 90% of repolarization (APD90) among conditions. The results are presented as the means ± SD of 3 independent experiments. * *P < 0.01 and * * *P < 0.001 versus the control group.
